# Efficacy and safety in synchronous core-needle biopsy and cryoablation for highly suspicious malignant pulmonary nodule

**DOI:** 10.1186/s40644-025-00901-0

**Published:** 2025-06-21

**Authors:** Tongyin Zhang, Qiaoyu Xu, Yuwan Hu, Haoyu Li, Haoran Du, Zhenguo Huang, Sheng Xie, Meng Yang, Yanyan Xu, Hongliang Sun

**Affiliations:** 1 Department of Radiology, China-Japan Friendship Hospital, No.2 Yinghua East Street, Chaoyang District, Beijing, 100029 China; 2 Graduate School, Chinese Academy of Medical Science &Peking Union Medical College, Beijing, 100730 China; 3 Department of Radiology, Beijing Chao-Yang Hospital, Capital Medical University, #8 Gong-Ti South Road, Chaoyang District, Beijing, 100020 China; 4 Peking University China-Japan Friendship School of Clinical Medicine, Beijing, 100029 China; 5 National Center for Respiratory Medicine, State Key Laboratory of Respiratory Health and Multimorbidity, National Clinical Research Center for Respiratory Diseases, Institute of Respiratory Medicine, Department of Pulmonary and Critical Care Medicine, Center of Respiratory Medicine, Chinese Academy of Medical Sciences, China-Japan Friendship Hospital, Beijing, 100029 China

**Keywords:** Core needle biopsy, Cryoablation, Computed tomography, Malignant pulmonary nodule

## Abstract

**Background:**

Percutaneous computed tomography (CT)-guided biopsy and cryoablation are commonly used techniques for diagnosing and treating pulmonary malignant tumors. Performing these procedures simultaneously allows for tissue diagnosis while potentially offering therapeutic benefits. This study aimed to evaluate whether the efficacy and safety of simultaneous percutaneous CT-guided biopsy and cryoablation in managing pulmonary tumors suspected of malignancy are comparable to those of sequential procedures.

**Methods:**

This retrospective study involved 124 patients with 131 highly suspicious malignant pulmonary nodules. Patients either underwent synchronous percutaneous core-needle biopsy and cryoablation (Group A) or separately underwent these procedures (Group B) from December 2020 to May 2024. All procedures were performed under CT guidance using a percutaneous approach. We analyzed technical success rates, complications, diagnostic yield, and local tumor control.

**Results:**

Technical success rates were 100% in both groups. The rate of pneumothorax was 42.1% (16/38) in Group A and 34.9% (30/86) in Group B. In Group A, hemoptysis and pleural effusion rates were 18.4% (7/38) and 23.7% (9/38), respectively, while in Group B, these rates were 16.3% (14/86) and 12.8% (11/86). These differences were not statistically significant. The diagnostic positive rate in Group A was 87.5%. The mean follow-up duration was 11.8 months (95% confidence interval [CI], 10.2–13.4), with local tumor control rates of 97% for Group A and 88% for Group B. The effectiveness rates of synchronous and separate procedures were similar.

**Conclusion:**

Synchronous biopsy-ablation is an effective method for obtaining tumor pathology and local treatment of lung tumors simultaneously. It is a viable option for select patients where expedited diagnosis-therapy is clinically justified, particularly when molecular profiling is not immediately indicated.

## Background

Pulmonary malignant tumors pose a significant health burden worldwide [1]. With the increasing use of low-dose computed tomography (CT) in lung cancer screening, the detection of pulmonary nodules has become more prevalent [2]. Accurate diagnosis and effective treatment are crucial for improving patient outcomes. Although surgical resection is the preferred treatment for lung cancer, many patients are not suitable candidates for surgery in clinical practice [3–5].

Percutaneous CT-guided biopsy and cryoablation are two widely utilized techniques in the management of pulmonary malignant tumors. CT-guided biopsy has been established as a safe method for obtaining tissue samples for histopathological examination, enabling accurate diagnosis and tumor classification [6, 7]. Cryoablation, on the other hand, is a minimally invasive treatment option that employs extreme cold temperatures to destroy tumor cells while also inducing a greater post-ablation immune response. This makes it a valuable alternative treatment for selected patients who are not suitable for surgery or other standard therapies [8–10].

For patients with a high suspicion of malignant pulmonary nodules, cryoablation can be performed based on imaging findings and tumor histories, without waiting for pathological results when the two are consistent. However, if the clinical diagnosis is uncertain or if evidence of gene mutations is needed for further treatment, a percutaneous biopsy becomes necessary. By combining these procedures, patients can achieve a tissue diagnosis while also receiving potential therapeutic benefits in a single session. This integrated approach has the potential to streamline the diagnostic and treatment process, reducing the need for multiple interventions and minimizing patient discomfort. While separate biopsy followed by cryoablation is standard for lesions requiring extensive immunologic/molecular profiling (e.g., non-small cell lung cancer needing targeted therapy). Despite the potential advantages, few studies have investigated the efficacy and safety of these synchronous procedures. Therefore, our study aims to assess whether the efficacy and safety of simultaneous percutaneous CT-guided biopsy combined with cryoablation are equivalent to those of sequential procedures.

## Materials and methods

### Study design and patient selection

Our study design was approved by the ethics review committee of China-Japan Friendship Hospital. All patients provided written informed consent to undergo simultaneous percutaneous CT-guided biopsy and cryoablation as part of their clinical care. This retrospective pilot study included 124 patients from December 2020 to May 2024. Patients who underwent percutaneous core-needle biopsy and cryoablation simultaneously were classified as Group A, whereas those who had these procedures performed separately were classified as Group B, with an interval of more than one week between the core-needle biopsy and cryoablation. The pathology was obtained by biopsy prior to cryoablation in these Group B patients. The inclusion criteria were as follows: Patients with pulmonary nodules ≤ 3 cm that were highly suspicious or already confirmed as malignant, indicated by signs such as lobulation, pleural indentation, bronchial inflation, vascular bundle involvement, or diameter enlargement during follow-up. These assessments were confirmed by multidisciplinary teams from the thoracic, respiratory, pathology, and radiology departments [11, 12]. Both core-needle biopsy and cryoablation had to be performed either synchronously or separately. Patients were deemed unsuitable for surgery due to multiple previous surgeries, advanced age, impaired lung function, or personal refusal. Exclusion criteria included: Severe coagulation disorders and an extremely poor health condition that prevented cooperation with medical staff.

### Technique procedures

Prior to the procedure, a pre-procedural CT scan was conducted to evaluate tumor characteristics and plan the cryoablation approach. This assessment included tumor size, location, proximity to critical structures, and the determination of the number of cryoablation probes required. Anticoagulant or antiplatelet medications were discontinued before the operation. Patients were positioned in the prone, supine, or lateral position, depending on the lesion’s location. The procedure was performed by two experienced interventional radiologists, each with 10 to 15 years of experience, using a 16-detector-row scanner (Aquilion 16; Canon Medical Systems) and argon and helium gas as cryogens. The technical parameters included: (1) scanning method: helical acquisition mode; (2) tube currents: automatic tube current modulation; (3) tube voltage: 120 kVp; and (4) slice thickness: 4–5 mm. Only the lesion area was scanned to minimize radiation exposure.

**Fig. 1:**
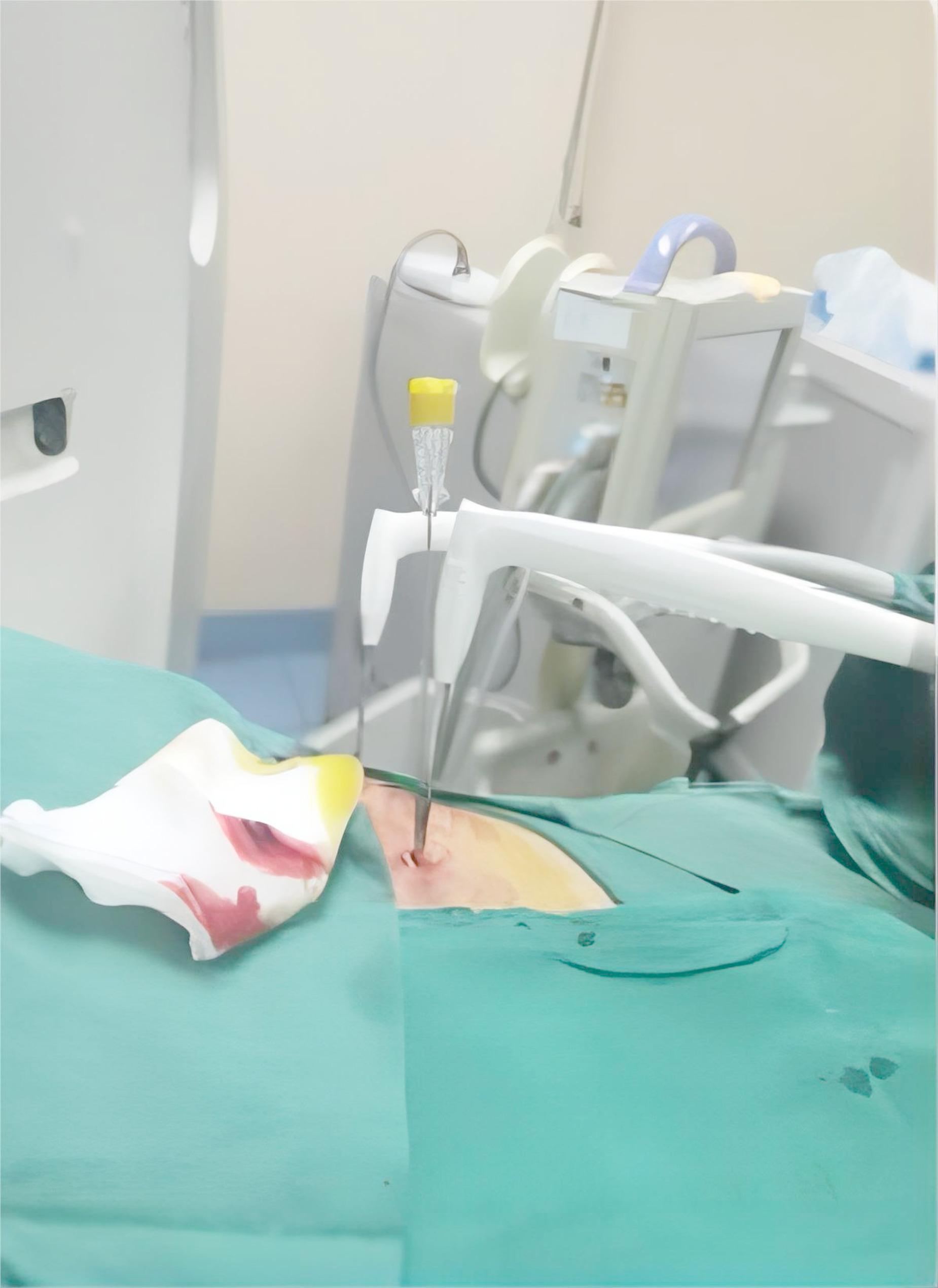
Synchronous biopsy and cryoablation procedure. The yellow needle is the core biopsy instrument, and two white needles are cryoablation probes

The biopsy system consisted of an introducer needle and an 18G or 20G core biopsy instrument (Argon Medical Devices, Inc., Athens, TX). The operators selected the appropriate approach to the lesion based on imaging to ensure the shortest puncture pathway while avoiding critical structures such as large blood vessels, bronchi, and interlobar fissures. Local pleural anesthesia was administered using 5 ml of 1% lidocaine. A 6.8 cm-long, 1.7 mm-diameter percutaneous introducer needle was advanced from the marked skin point, ensuring it did not penetrate the parietal pleura. Once the needle was positioned within the target lesion, two to three specimens were collected using the core biopsy instrument for subsequent histological evaluation and diagnosis. Before cryoablation, we performed a preoperative integrity test of the cryoprobe in a saline solution to confirm its functionality and rule out the possibility of argon leakage. Cryoprobes (Endocare, Irvine, California) were then inserted into the target area parallel to the lesion, maintaining a distance of less than 0.5 cm between the probe and the nodule’s edge (Fig. 1). A double-probe method was employed in cases where a single probe could not achieve complete ablation. This technique created a “clamping freezing” effect, allowing both cryoprobes to deliver energy from either side of the tumor, ensuring comprehensive coverage by the ablation zone for optimal results [13].

**Fig. 2:**
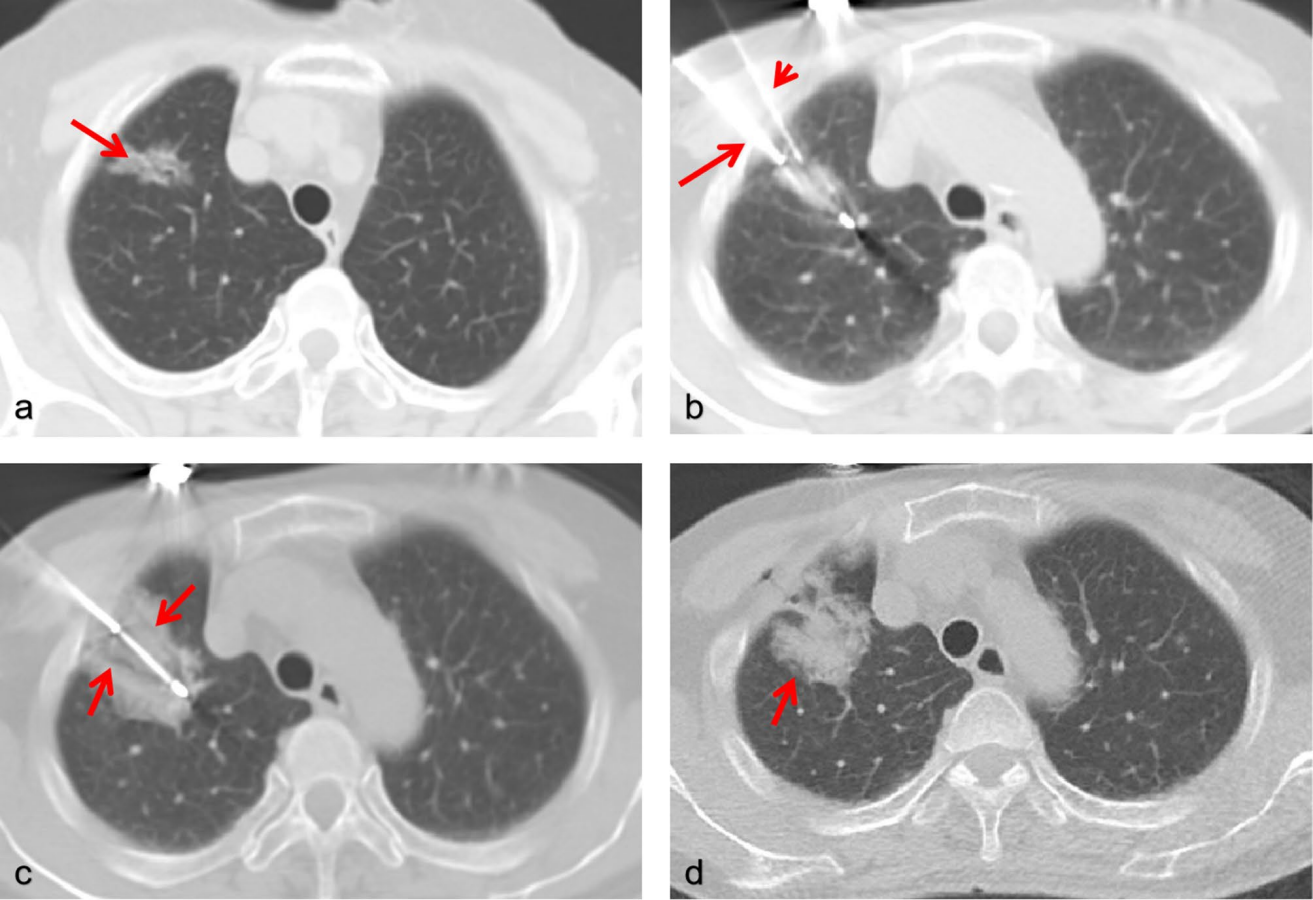
Synchronous procedure in a 71-year old woman. (**a**) Computed tomography image showing a single sub-solid nodule (arrow), 20 mm ×12 mm, in the apical segment of the right upper lobe, irregular margin, and pleural tail sign. (**b**) A cryoablation probe (long arrow) was placed into the nodule while the biopsy needle was at the edge of the lesion (short arrow). (**c**) During the cyroablation procedure, the locus center began to occur liquefactive necrosis as well as the ice ball emerged (arrows). (**d**) In the thoracic computed tomography scan immediately after cryoablation, the ablated nodule presented with a circular ground-glass opacity (arrow). Finally, the pathological result showed it an early stage lung adenocarcinoma

**Fig. 3:**
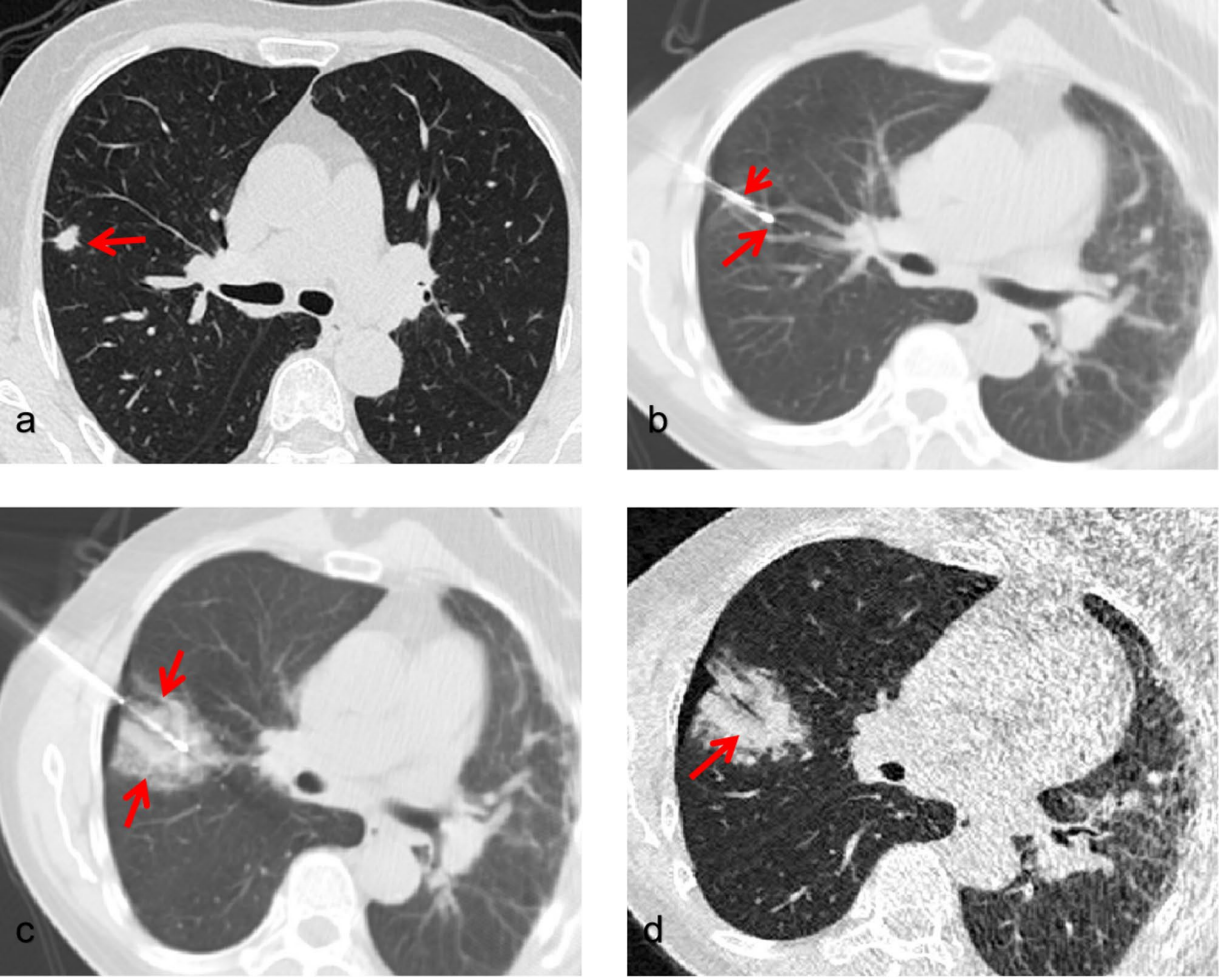
Synchronous procedure in a 77-year old woman. (**a**) Computed tomography image showing a single solid nodule (arrow), 11 mm ×6 mm, in the apical segment of the right superior lobe, regular margin, with pleural tail sign. (**b**) A cryoablation probe (long arrow) was placed into the nodule while the biopsy needle was at the edge of the lesion (short arrow). (**c**) During the cyroablation procedure, the locus center began to occur liquefactive necrosis as well as the ice ball emerged (arrows). (**d**) In the thoracic computed tomography scan immediately after cryoablation, the ablated nodule presented with a circular ground-glass opacity (arrow). Eventually, the pathological result showed it a lung adenocarcinoma

Cryoablation involved rapidly cooling the tissue using argon gas, which formed an ice ball around the tumor [14]. Two or three freezing-thawing cycles were applied, with each freezing cycle lasting 10 to 15 min, interspersed with a five-minute heating session with helium. The total freezing time was 30 min. Real-time CT imaging was performed every five minutes to assess the ice ball’s formation. The edge of the ice ball was maintained at least 1.0 cm larger than the tumor’s edge to ensure complete ablation and satisfactory clinical outcomes (Figs. 2 and 3).

In Group B, patients diagnosed with primary lung cancer or metastases after biopsy subsequently underwent percutaneous cryoablation once any complications from the biopsy were resolved. The technical aspects of the biopsy and cryoablation in this group were similar to those in the synchronous procedure.

### Post-procedural care and follow-up

After cryoablation, patients were monitored in a recovery area for a specified period. Post-procedural CT scans were conducted to assess the immediate technical success of the cryoablation procedure, ensuring that the edge of the ice ball was 0.5–1 cm larger than the tumor’s edge to confirm complete ablation. These scans also aimed to detect any immediate complications, such as pneumothorax, hemorrhage, pleural effusion, and other adverse events [15].

Treatment-related complications were evaluated according to the Society of Interventional Radiology criteria [16, 17]. The primary complications included pneumothorax, hemoptysis, and pleural effusion. Major complications are life-threatening and necessitate prolonged hospitalization, while minor complications are self-limiting and typically require only a short hospital stay for observation or treatment.

The degree of pneumothorax was classified as severe (lung compression > 50%), moderate (lung compression ≤ 50% and > 20%), and mild (lung compression ≤ 20%). Hemoptysis was categorized as severe (> 100 mL), moderate (10–100 mL), and mild (≤ 10 mL). Pleural effusion was classified as severe (> 1000 mL), moderate (500–1000 mL), and mild (≤ 500 mL).

Follow-up observations included pathological results, the technical success rate, and the local tumor control rate. Local tumor progression (LTP) was identified by an increase in the ablation zone size compared to the baseline CT scan performed one month after cryoablation, accompanied by irregular, scattered, nodular, or eccentric enhancement, or circumferential expansion during imaging follow-up [18].

### Data collection and statistical analysis

Demographic data of the enrolled patients, characteristics of pulmonary nodules, procedural details, complications, and efficacy rates per nodule were collected for statistical analysis. All parameter values were tested for normality and homogeneity of variance. Continuous variables were expressed as mean ± standard deviation for normally distributed data and median ± interquartile range for non-normally distributed data. Differences between the synchronous and separate procedures were analyzed using Pearson’s Chi-squared test or Fisher’s exact test for categorical variables. A p-value of < 0.05 was considered statistically significant. All analyses were conducted using SPSS software version 25.0.

## Results

### Basic clinical characteristics

The basic characteristics of the eligible studies and patients are summarized in Table 1. Group A included thirty-eight patients with 40 highly suspicious malignant pulmonary nodules who underwent synchronous core-needle biopsy and cryoablation via a coaxial introducer. This group comprised 36 patients with a single nodule and 2 patients with 2 nodules each. Group B included eighty-six patients with 91 pathologically proven malignancies identified by core needle biopsy or who separately underwent cryoablation; this group consisted of 81 patients with a single nodule and 5 patients with 2 nodules each.

**Table 1:** Basic clinical data of patients

Characteristic	Group A	Group B	*P*
Patient number	38	86	
Sex(males)	19/38	47/86	0.632
Sex(females)	19/38	39/86	
Age (years, mean ± SD)^*^	65.37 ± 12.73	65.91 ± 10.93	0.437
Cancer history	12/38	46/86	0.024
Lung cancer	7	13	
Colorectal cancer	3	20	
Ovarian cancer	1	1	
Breast cancer	1	4	
Cervical cancer	0	2	
Renal cancer	0	2	
Bladder cancer	0	1	
Gastric cancer	0	2	
Testicular cancer	0	1	
Underlying pulmonary disease	8/38	22/86	0.587
Emphysema	5	18	
Interstitial lung disease	5	9	

Continuous data are presented as mean ± standard deviation or mean ± interquartile range (^*^); Categorical data were expressed with frequencies/ total patient numbers of its group

In terms of underlying pulmonary conditions, 8 patients (12.1%) in Group A and 22 patients (25.6%) in Group B had conditions such as emphysema or interstitial lung disease. Additionally, 12 patients (31.6%) in Group A and 46 patients (53.4%) in Group B had a history of malignant tumors.

### Procedure and complications

Tables 2 and 3 summarize the characteristics of the procedures and associated complications. All synchronous and separate procedures were successfully completed, achieving a technical success rate of 100% in both groups. Pneumothorax was the most common complication, with incidences of 42.1% in Group A and 34.9% in Group B. Patients with mild pneumothorax generally recovered spontaneously after conservative management. Only two patients in each group needed chest tube drainage.

**Table 2:** Basic data of the nodule and procedure

Characteristic	Group A	Group B	*P*
Number of nodules	40	91	
Number of nodules per patient			
1	36	81	
2	2	5	
Tumor location			0.457
Right upper lobe	12/40	31/91	
Right middle lobe	0/40	4/91	
Right lower lobe	11/40	23/91	
Left upper lobe	9/40	15/91	
Left lower lobe	8/40	18/91	
Patients’ position			0.833
Supine	11/38	21/86	
Prone	1738	43/86	
Lateral	10/38	22/86	
Tumor types			0.396
solid	21/40	55/91	
sub-solid	19/40	36/91	
Maximum axial tumor diameter^*^ (cm)	1.83 ± 0.73	1.46 ± 0.70	0.392
Depth of lung nodule from pleural surface(cm)	4.20 ± 1.09	4.45 ± 1.53	0.349
Specs of the biopsy needle			
18G/20G	6/34		

Continuous data are presented as mean ± standard deviation or mean ± interquartile range (*); Categorical data were expressed with frequencies/ total patient numbers of its group

**Table 3:** Complication of procedures

	Group A	Group B	*P*
Pneumothorax (%)	42.1	34.9	0.502
Mild	9/38	21/86	
Moderate	4/38	3/86	
Severe	3/38	6/86	
Needed chest tube drainage	2/38	2/86	
Pleural effusion (%)	23.7	12.8	0.128
Mild	6/38	9/86	
Moderate	1/38	2/86	
Severe	2/38	0/86	
Hemoptysis (%)	18.4	16.3	0.769
Mild	6/38	8/86	
Moderate	1/38	6/86	
Severe	0/38	0/86	
Air embolism	0	0	
Bronchopleural fistula	0	0	

Continuous data are presented as mean ± standard deviation. Categorical data were expressed with frequencies/ total patient numbers of its group

Notably, hemoptysis occurred in 18.4% of patients in Group A and 16.3% in Group B. The incidence of pleural effusion was 23.7% in Group A and 12.8% in Group B. Patients who experienced sudden hemoptysis during the biopsy were encouraged to cough gently and expel the blood. Those with hemoptysis were positioned laterally with the punctured side down, while careful observation and intravenous administration of hemostatic medications were administered. Rare complications, such as air embolization, bronchopleural fistula, pulmonary artery pseudoaneurysm, and tumor needle seeding, were not detected during the follow-up period [19, 20]. There was no statistically significant difference in the incidence rates of complications between the two groups. Importantly, no patients died following the procedures.

### Pathological findings

The histopathological results of the biopsy specimens were re-analyzed by two experienced pathologists, each with 15 years of experience. The assessment process involved an initial evaluation by one pathologist, followed by a review by the second pathologist to reach a consensus, ensuring the reliability of the results. Upon analyzing the 131 nodules from 124 patients, 126 nodules were diagnosed as malignant. In Group A, specimens obtained through CT-guided biopsy did not contain tumor cells; specifically, 5 cases were diagnosed as atypical inflammatory hyperplasia of epithelial cells, 1 case as inflammatory cell infiltration, and 4 cases as fibrous cells. The remaining nodules were confirmed as malignant upon pathological analysis, with the details of the biopsy specimen types provided in Table 4.

**Table 4:** Detailed pathological types of biopsy specimen

Pathologic result	Group A	Group B
Adenocarcinoma	29/40	52/91
Squamous cell carcinoma	3/40	8/91
SCLC	1/40	2/91
Metastasis	2/40	29/91
Chronic inflammation	1/40	0/91
Fibrous cells	4/40	0/91

SCLC: small cell lung cancer

### Efficacy analysis

A total of 101 patients continued to be observed, with a mean follow-up duration of 11.8 months (95% confidence interval [CI], 10.2–13.4). All patients underwent unenhanced CT scans at one month, three months, and six months post-cryoablation, followed by scans every six months thereafter. Tumor progression was noted in one patient in Group A and nine patients in Group B. Importantly, no procedure-related or tumor-related deaths occurred during the follow-up period. The effectiveness rates of the synchronous and separate procedures were not statistically different.

## Discussion

In recent years, image-guided percutaneous lung ablation has significantly advanced and is emerging as a promising treatment option due to its minimally invasive nature, fewer complications, substantial efficacy, and rapid recovery. This technique helps preserve patients’ pulmonary function as much as possible [21]. However, the simultaneous performance of percutaneous CT-guided biopsy and cryoablation has rarely been reported in the literature. This combined approach has the potential to avoid unnecessary therapies and facilitate appropriate adjuvant treatments based on pathological findings. This retrospective study aims to enhance existing knowledge by providing evidence on the feasibility and outcomes of this combined method. In our study, the technical success rate was 100%, indicating the effectiveness of CT-guided cryoablation in achieving complete tumor ablation. The diagnostic yield of the simultaneous percutaneous CT-guided biopsy was 87.5%, demonstrating successful tissue diagnosis. This suggests that the combined procedure effectively obtained adequate tissue samples for pathological examination.

Pneumothorax was the most common complication associated with either percutaneous biopsy or cryoablation [22]. It was more likely to occur in patients with two nodules or those with a single lesion but treated with dual-needle ablation. In addition, it was also prone to occur in patients with underlying lung conditions such as emphysema and interstitial lung disease. Although the rates between the two groups did not show a statistical difference, repeat pleural punctures can increase the risk of pneumothorax [23, 24]. Notably, pneumothorax occurred in over 40% of all procedures, underscoring the inherent risks of percutaneous lung intervention regardless of timing. This highlights the critical importance of patient selection, operator expertise, and readiness for complication management. Hemoptysis is typically the second most common complication of lung cryoablation, occurring more frequently than with radiofrequency or microwave ablation [25, 26]. This complication may be associated with factors such as lesion location, blood supply, and the number of cryoablation probes used. The pathological cause of hemoptysis is primarily pulmonary vascular injury resulting from tissue damage. However, there was no statistically significant difference in the incidence rates of hemoptysis between the two groups, suggesting that the combined procedures may not increase the risk. Furthermore, previous studies have indicated that active electrical thawing after the final freeze in a triple-freeze protocol can reduce both the incidence and severity of hemoptysis, as well as delay its onset following percutaneous cryoablation [27]. Our data suggest that although the synchronous procedure increased the number of punctures, the incidence of pleural effusion did not rise. The lack of statistical difference in complications between the two groups may also be related to the fact that the biopsy needles selected in the simultaneous biopsy-ablation group were mostly 20-gauge, which are finer and relatively safer. Previous case reports have pointed out certain fatal risks associated with cryoablation, such as air embolism [28]. To mitigate this risk, we performed a preoperative integrity test of the cryoprobe in a saline solution to confirm its functionality and rule out the possibility of argon leakage. Then, during the cryoablation procedure, we rapidly cooled the tissue using argon gas, which effectively created an ice ball around the tumor, significantly lowering the chance of air embolism.

Five nodules were not pathologically diagnosed as malignant lesions; however, core needle biopsy was still deemed necessary to clarify the diagnosis. Additionally, malignancy could not be fully excluded due to the potential for false-negative results, as the imaging manifestations and clinical follow-up already suggested malignancy. Consequently, the patients opted for aggressive management. The synchronous cryoablation not only aimed to inactivate potential malignant cells but also helped alleviate patient anxiety. These five patients were advised to attend follow-up appointments every six months during the first year after the procedure to mitigate the risk of false-negative results delaying further treatment. All patients agreed to long-term follow-up to monitor for disease relief or progression. Although synchronous biopsy-cryoablation can establish a definitive diagnosis, this approach is not suitable for lesions requiring upfront comprehensive molecular profiling (e.g., actionable mutations, PD-L1). Synchronous cryoablation may compromise tissue availability for such testing; thus, patient selection is critical. Our cohort primarily included patients with contraindications to surgery or systemic therapy, where rapid local control outweighed the need for extensive immunologic data. While, synchronous biopsy-ablation addresses niche clinical needs: (1) High-surgical-risk patients with strong radiologic malignancy suspicion (e.g., PET-avid, serial growth) where immediate local control is prioritized over molecular data. (2) Peripheral nodules with low complication risk, where biopsy yields adequate tissue for histologic diagnosis (e.g., confirming NSCLC vs. metastasis) but molecular profiling is deferred due to patient comorbidities or palliative intent. (3) Logistical constraints (e.g., limited healthcare access, inability to tolerate multiple procedures, shorten the length of hospital stay and reduce hospitalization costs). Three nodules diagnosed as small cell lung cancer (SCLC) were ablated. According to the NCCN guidelines for non-small cell lung cancer (NSCLC), the recommended treatment for SCLC is chemotherapy, and there is insufficient evidence to support the use of cryoablation as a standard treatment. However, it may serve as an adjunctive therapy to reduce tumor activity [29, 30]. More research is needed to establish its efficacy. The effectiveness rates of synchronous and separate procedures were not statistically different, indicating that both approaches are effective in achieving local tumor control.

The limitations of this study include its single-center design. Multi-center studies involving larger patient populations would provide a more comprehensive evaluation of the effectiveness and safety of this combined approach. Furthermore, this approach is not suitable for lesions requiring upfront comprehensive molecular profiling (e.g., actionable mutations, PD-L1). Thus, the study population may not be representative of all patients with pulmonary malignant tumors, The short follow-up duration may restrict the assessment of long-term outcomes and complications. These limitations should be taken into account when interpreting the results. Future studies could focus on larger patient cohorts, longer follow-up periods, and comparative analyses with alternative treatment approaches. Additionally, investigations aimed at optimizing procedural techniques, refining patient selection criteria, and enhancing post-procedural care protocols would be valuable for improving the outcomes of simultaneous percutaneous CT-guided biopsy and cryoablation.

## Conclusion

Synchronous CT-guided biopsy and cryoablation is technically feasible with comparable complication rates to separate procedures in highly selected patients. The high diagnostic yield and technical success rate, along with favorable local tumor control, support the feasibility of this combined method. However, it should not replace sequential workflows when histologic/immunologic characterization is essential.

## Data Availability

No datasets were generated or analysed during the current study.

## Abbreviations

CT: Computed tomography
NCCN: National Comprehensive Cancer Network
SCLC: Small cell lung cancer
NSCLC: Non-Small cell lung cancer
LTP: Local tumor progression
PD-L1: Programmed cell death ligand 1

## References

[1] Kratzer TB, et al. Lung cancer statistics, 2023. Cancer. 2024;130(8):1330–48. 38279776 10.1002/cncr.35128

[2] Rashidi A, et al. Lung cancer screening updates: impact of 2023 American Cancer society’s guidelines for lung cancer screening. Clin Imaging. 2024;113:110229. 38941769 10.1016/j.clinimag.2024.110229 PMC12619990

[3] Howington JA, et al. Treatment of stage I and II non-small cell lung cancer: diagnosis and management of lung cancer, 3rd ed: American college of chest physicians evidence-based clinical practice guidelines. Chest. 2013;143(5 Suppl):eS278–313. 10.1378/chest.12-2359 23649443

[4] Winckelmans T, et al. Segmentectomy or lobectomy for early-stage non-small-cell lung cancer: a systematic review and meta-analysis. Eur J Cardiothorac Surg. 2020;57(6):1051–60. 31898738 10.1093/ejcts/ezz339

[5] Nogrady B. Research round-up: lung cancer. Nature. 2020;587(7834):S8–9. 33208968 10.1038/d41586-020-03153-z

[6] Wang J, et al. Comparison between percutaneous transthoracic co-axial needle CT-guided biopsy and transbronchial lung biopsy for the diagnosis of persistent pulmonary consolidation. Insights Imaging. 2023;14(1):80. 37166531 10.1186/s13244-023-01436-3 PMC10175526

[7] Heerink WJ, et al. Complication rates of CT-guided transthoracic lung biopsy: meta-analysis. Eur Radiol. 2017;27(1):138–48. 27108299 10.1007/s00330-016-4357-8 PMC5127875

[8] Tian Y, et al. Cryoablation and immune synergistic effect for lung cancer: A review. Front Immunol. 2022;13:950921. 36389781 10.3389/fimmu.2022.950921 PMC9647087

[9] Aarts BM, et al. Cryoablation and immunotherapy: an overview of evidence on its synergy. Insights Imaging. 2019;10(1):53. 31111237 10.1186/s13244-019-0727-5 PMC6527672

[10] Palussiere J, et al. Primary tumors of the lung: should we consider thermal ablation as a valid therapeutic option? Int J Hyperth. 2019;36(2):46–52. 10.1080/02656736.2019.1647351 31537155

[11] Mazzone PJ, Lam L. Evaluating the patient with a pulmonary nodule: A review. JAMA. 2022;327(3):264–73. 35040882 10.1001/jama.2021.24287

[12] Gould MK et al. Evaluation of individuals with pulmonary nodules: when is it lung cancer? Diagnosis and management of lung cancer, 3rd ed: American College of Chest Physicians evidence-based clinical practice guidelines. Chest, 2013. 143(5 Suppl): p. e93S-e120S. 10.1378/chest.12-2351 PMC3749714 23649456

[13] Kamaruzaman N et al. Functionalised hybrid Collagen-Elastin for acellular cutaneous substitute applications. Polym (Basel), 2023. 15(8). 10.3390/polym15081929 PMC10143773 37112076

[14] Amoils SP. The joule Thomson cryoprobe. Arch Ophthalmol. 1967;78(2):201–7. 4952598 10.1001/archopht.1967.00980030203014

[15] Wang X, et al. Margin size is an independent predictor of local tumor progression after ablation of colon cancer liver metastases. Cardiovasc Intervent Radiol. 2013;36(1):166–75. 22535243 10.1007/s00270-012-0377-1 PMC4122121

[16] Genshaft SJ, et al. Society of interventional radiology quality improvement standards on percutaneous ablation of Non-Small cell lung Cancer and metastatic disease to the lungs. J Vasc Interv Radiol. 2021;32(8):e12421–124210. 10.1016/j.jvir.2021.04.027 34000388

[17] Khalilzadeh O, et al. Proposal of a new adverse event classification by the society of interventional radiology standards of practice committee. J Vasc Interv Radiol. 2017;28(10):1432–e14373. 28757285 10.1016/j.jvir.2017.06.019

[18] Ahmed M, et al. Image-guided tumor ablation: standardization of terminology and reporting criteria–a 10-year update. J Vasc Interv Radiol. 2014;25(11):1691–705. e4. 25442132 10.1016/j.jvir.2014.08.027 PMC7660986

[19] Sachdeva M, et al. Complications and yield of computed Tomography-Guided transthoracic core needle biopsy of lung nodules at a High-Volume academic center in an endemic coccidioidomycosis area. Lung. 2016;194(3):379–85. 26980483 10.1007/s00408-016-9866-3

[20] Wang Y, et al. CT-guided percutaneous transthoracic needle biopsy for paramediastinal and nonparamediastinal lung lesions: diagnostic yield and complications in 1484 patients. Med (Baltim). 2016;95(31):e4460. 10.1097/MD.0000000000004460 PMC4979835 27495081

[21] Shimizu K, et al. Percutaneous CT-guided fine needle aspiration for lung cancer smaller than 2 cm and revealed by ground-glass opacity at CT. Lung Cancer. 2006;51(2):173–9. 16378659 10.1016/j.lungcan.2005.10.019

[22] Alzubaidi SJ et al. Percutaneous Image-Guided ablation of lung tumors. J Clin Med, 2021. 10(24). 10.3390/jcm10245783 PMC8707332 34945082

[23] Huo YR, et al. Pneumothorax rates in CT-Guided lung biopsies: a comprehensive systematic review and meta-analysis of risk factors. Br J Radiol. 2020;93(1108):20190866. 31860329 10.1259/bjr.20190866 PMC7362905

[24] Ruud EA, et al. Predictors of pneumothorax and chest drainage after percutaneous CT-guided lung biopsy: A prospective study. Eur Radiol. 2021;31(6):4243–52. 33354745 10.1007/s00330-020-07449-6

[25] McDevitt JL, et al. Percutaneous cryoablation for the treatment of primary and metastatic lung tumors: identification of risk factors for recurrence and major complications. J Vasc Interv Radiol. 2016;27(9):1371–9. 27321886 10.1016/j.jvir.2016.04.005

[26] Palussiere J, Catena V, Buy X. Percutaneous thermal ablation of lung tumors - Radiofrequency, microwave and cryotherapy: where are we going? Diagn Interv Imaging. 2017;98(9):619–25. 28844613 10.1016/j.diii.2017.07.003

[27] Wrobel MM, et al. Active versus passive thaw after percutaneous cryoablation of pulmonary tumors: effect on incidence, grade, and onset of hemoptysis. AJR Am J Roentgenol. 2021;217(5):1153–63. 34008999 10.2214/AJR.21.25872

[28] Sandomirsky M, Crifasi JA, Long C, Mitchell EK. Case report of fatal complication in prostatic cryotherapy. First reported death due to argon gas emboli. Am J Forensic Med Pathol. 2012;33(1):68–72. 22442836 10.1097/paf.0b013e3181dd5b8c

[29] Ettinger DS, et al. NCCN Guidelines(R) insights: Non-Small cell lung cancer, version 2.2023. J Natl Compr Canc Netw. 2023;21(4):340–50. 37015337 10.6004/jnccn.2023.0020

[30] Blackhall F, et al. Treatment patterns and outcomes among patients with small-cell lung cancer (SCLC) in europe: a retrospective cohort study. BMJ Open. 2023;13(2):e052556. 36746549 10.1136/bmjopen-2021-052556 PMC9906168

